# Robot-Assisted Surgery: Current Applications and Future Trends in General Surgery

**DOI:** 10.7759/cureus.82318

**Published:** 2025-04-15

**Authors:** Juan Carlos Rojas Burbano, Nicolás Idárraga Ruiz, Gustavo Andrés Rojas Burbano, José Sebastián Guacho Inca, Carlos Alejandro Arragan Lezama, Manuel Sánchez González

**Affiliations:** 1 General Medicine, Pontificia Universidad Católica del Ecuador, Quito, ECU; 2 Surgery, Clínica Medicádiz, Ibagué, COL; 3 Surgery, Hospital de Especialidades Eugenio Espejo, Quito, ECU; 4 General Practice, Universidad Anáhuac Xalapa, Veracruz, MEX; 5 Surgery, Hospital de Especialidades, Centro Médico Nacional de Occidente, IMSS, Guadalajara, MEX

**Keywords:** applications and trends, general surgery, literature review, robot-assisted surgery, robot-assisted surgery (ras)

## Abstract

Advancements in digital equipment have been applied to treating complex medical cases. However, the literature has not comprehensively reported these applications, and the outcomes of conventional surgeries have not been compared with robot-assisted surgeries to determine efficacy. Therefore, this literature review aimed to find and compare outcomes. According to existing literature, robot-assisted surgery has brought significant changes to various aspects of general surgical practice. The research outcomes have shown that robotic surgery minimizes surgical invasiveness by enabling smaller incisions, reducing blood loss, and accelerating patient recovery. The da Vinci Surgical System has shown enhanced surgical accuracy due to better control when operating in complex cases such as colorectal resections, pancreaticoduodenectomy, and hernia repairs. The use of robotic systems enables surgeons to remain comfortable while performing complex procedures because the system allows them to maintain ergonomic body positions. Robotic systems lead to better clinical outcomes for patients, such as rapid hospital discharge, fewer complications, and faster recovery times. The advantages of robotic surgery will become more apparent once technical challenges regarding costs, learning curves, and haptic feedback are fully addressed.

## Introduction and background

Modern general surgery has been transformed by the introduction of robot-assisted surgery (RAS) technology, which has revolutionized the execution of complex procedures. Advancements in technology and robotics have allowed medical professionals to combine precision with flexible surgical approaches, resulting in improved patient outcomes through RAS [1]. This system originated from the development of minimally invasive surgery (MIS). Through advanced surgical methods, surgeons gain full control over demanding procedures while effectively treating complex anatomical areas and minimizing human errors.

RAS provides various healthcare benefits, such as reducing blood loss and trauma, faster recovery times, smaller surgical incisions to reduce complications, and shorter hospitalization stays [2]. This system implements the following two essential elements: the surgeon’s master console, which displays the surgical field three-dimensionally with enhanced magnification, and robotic arms that perform precise, surgeon-controlled movements [3]. Microjointed robotic arms enable precise movements of surgical instruments due to their advanced joint technology. The master-slave system provides the surgeon with full control of the robotic arms, improving surgeons’ capabilities while maintaining their expert role [4].

The da Vinci Surgical System (Mountain View, CA: Intuitive Surgical, Inc.) is a well-known robotic system that is frequently used in general surgery. Other innovative robotic solutions include the Senhance Surgical System (Durham, NC: TransEnterix) and the Versius Surgical Robotic System (Cambridge, UK: CMR Surgical Ltd.) [5,6]. These robotic systems work in tandem with surgeons to enhance ergonomics, reduce fatigue, and increase precision in both basic laparoscopic techniques and more complex gastrointestinal and colorectal surgical procedures.

RAS serves as a high-tech appendage that extends the capabilities of surgeons, enabling precise and refined movements. As robotic technology continues to advance, its potential in general surgical operations grows, leading to safer and more efficient surgical procedures [4]. This review examines the utilization of robot-integrated surgery in general practice, evaluating its current application in minimally invasive colorectal and hernia repairs and predicting future trends that could further improve surgical practices.

Scope of the review

A comprehensive research scope must be established to enable a comprehensive examination and thorough coverage of the subject matter. This review explores RAS via the following three essential points: its historical advancement, its current uses in general surgery, and trends expected for future development.

An exploration of RAS’s historical development will follow its evolution from early mechanical surgical tools to modern robotic systems. The historical evolution of robotic surgery allows us to follow major technological advancements and recognize the leading figures who have contributed to the evolution of medical practices over the decades. The discussion will focus on the present utilization of RAS within general surgical practices. The section examines how robotic systems have been implemented in general surgical procedures, including laparoscopic surgeries, hernia repairs, and gastrointestinal procedures. It also assesses the strengths of robotic surgery applications, particularly focusing on precision, rapid recovery time, and better patient-related outcomes, while acknowledging financial aspects and the training needs of surgical staff. The future of RAS will be examined through analysis of emerging technological developments, including artificial intelligence, machine learning, and advanced imaging capabilities. The analysis will also extend to projected applications of robotics systems across new surgical fields and the integration of telemedicine and ethical considerations related to progressively automated surgical robots.

This review aimed to identify and clarify major research concepts that are emerging from existing scientific publications. It comprises research on the safety and effectiveness of robotic interventions compared to conventional methods (such as open surgery or non-robotic surgeries) as well as studies on the impact of robotic technology on surgical training, educational methods, and healthcare implications for patient access. This literature review uses a structured approach to investigate RAS both in the contemporary era and for the future.

## Review

United States Defense Advanced Research Projects Agency (DARPA)

RAS was identified as a concept in the 1980s, with funding from NASA and the United States Defense Advanced Research Projects Agency (DARPA) for the development of remote surgical systems for hostile military and space environments [7,8]. The PUMA 560 surgical robot (Chicago, IL: Encyclopædia Britannica, Inc.) became the first robotic system to assist in neurosurgery by performing a biopsy in 1985​ [9]. From 1988 to 1994, medical technology evolved through innovations such as PROBOT (Bath, England: Active Robotics Ltd.) and the Automated Endoscopic System for Optimal Positioning (Goleta, CA: Computer Motion), which brought about a fundamental change to minimally invasive surgery with its teleoperated camera positioning system [7,10].

In 1999, the creation of the historic da Vinci Surgical System (Mountain View, CA: Intuitive Surgical, Inc.) marked a breakthrough in technology [11]. Medical professionals used the da Vinci system first in urological treatments, but its robotic arms, with improved dexterity and 3D high-definition imaging, allowed for highly accurate minimal-access surgeries​ [12]. Intuitive Surgical released three successive updates to the da Vinci platform between 2003 and 2014: S, followed by Si, and finally Xi [13]. Each new version added better technology capabilities, including enhanced flexibility, improved imaging, and dual-console operation for surgical teams.

RAS experienced rapid growth with the da Vinci system, and it successfully helped surgeons perform intricate procedures, such as cholecystectomies, colorectal surgeries, and hernia repairs, in general surgery. The precision enhancement offered by robotic surgery is one of the primary reasons for its success in general surgery because it enables surgeons to operate with improved control within confined anatomical spaces [14]. The robotic systems also include tremor-filtering technology that enhances surgical movement quality and reduces human error [15]. This technology provides exceptional precision during bariatric surgery and liver resection operations, both of these procedures require precise dissection [7].

The practice of robotic surgery moved past laparoscopic procedures starting in the early 2000s. This system currently enables pancreaticoduodenectomy and rectal cancer surgery procedures, marking a shift toward more advanced robotic surgical operations [12]. The influence of robotic surgery has even extended to emergency general surgery procedures. The adoption of robotic systems for cholecystectomy, colectomy, inguinal hernia, and ventral hernia repairs has become more common in emergency surgical settings [16]. The total cost of robotic surgical procedures has slowed their implementation, yet their enduring advantages of faster recovery time, lower complication risks, and heightened procedural precision are accelerating their current use [8]. The technology improvement that includes artificial intelligence (AI)-powered decision systems and predictive analyses will drive robotic surgery toward domination of general surgery practices. The key milestones of robot-assisted surgical procedures are mentioned in Table 1.

**Table 1:** Timeline of key milestones in robotic surgery.

Years	Key milestones
1980s	PUMA 560 and PUMA 200 (Chicago, IL: Encyclopædia Britannica, Inc.): the first surgical robots for neurosurgery [9].
	Neuromate (Wotton-under-Edge, England: Renishaw plc): a robot used in neurosurgery for stereotactic procedures, such as biopsies [9].
1990s	PROBOT (Bath, England: Active Robotics Ltd.): a modified industrial robot used for prostatectomy, introduced in the early 1990s [7,10].
	Zeus Systems 1990-2025 (Midland, Canada: Zeus Systems Inc.): first robotic system to introduce remote control (computer motion) for laparoscopy with multiple robotic arms [10].
1994	Robodoc (St. Louis, MO: Stereotaxis, Inc.): it is designed for orthopedic surgeries, such as hip arthroplasty [10].
1999	da Vinci Surgical System (Mountain View, CA: Intuitive Surgical, Inc.): first robotic system for general surgery. Intuitive Surgical introduced it through Zeus System, particularly used for laparoscopic surgery [11].
2001	da Vinci robotic system: approved by FDA [11,12].
2002-2014	Advancement in da Vinci robot system models 1999-2025 (Mountain View, CA: Intuitive Surgical, Inc.): S, Si, Xi models introduced with better precision and flexibility [13].
2010-Present	Robotic surgery: expanding into bariatric, colorectal surgeries [7,14,15].
	Robotic emergency procedures: growing rapidly, with growing popularity in developing countries [7,14,15].
2018	da Vinci SP 2025 (Mountain View, CA: Intuitive Surgical, Inc.): a single-port robotic system designed to reduce invasiveness using a single incision [13].
2021	Versius Surgical Robotic System 2025 (Cambridge, UK: CMR Surgical Ltd.): a flexible modular system with compact arms enhancing utilization across different surgical procedures [14,16].
2023	Robocath’s R-One 2018-2025 (Rouen, France: Robocath): a robotic platform for endovascular procedures to improve precision and reduce exposure to radiation [14,16].

Core technologies involved in robotic systems

RAS benefits substantially from the evolution of core robotic-system technologies used in minimally invasive robot-assisted surgical operations. Surgical robots linked with imaging technology through sensing platforms provide surgeons with better precision, enhanced stroke management, and the dexterity needed to perform complex, minimally invasive procedures. The primary technologies employed in robotic systems are mentioned below.

Robotic Arms with Manipulators

The robots serve as extensions of surgical tools, enabling surgeons to maintain better dexterity, flexibility, and enhanced stability during procedures. Robotic arms allow for precise instrument control because they perform movements accurately to minimize human error. These medical robots include specialized surgical tools such as scissors, staplers, and cauterizers, which support various procedures in MIS [17,18].

Master-Slave Teleoperation

A fundamental feature of robotic systems allows surgeons to control robotic arms through master-slave teleoperation using joysticks and foot pedals as input controls from their console. The slave configuration provides precise relocation of surgical instruments inside the human body, helping surgeons reduce manual tremors and boost operational precision [7,14].

3D Visualization

High-definition 3D cameras are an essential part of robotic systems, enabling surgeons to view surgical sites with better clarity. Robotic surgery’s primary advantage comes from a high-definition 3D camera system that enhances surgeons’ visibility during operations. Surgeons view internal structures in complete detail through cameras that connect to processing tools such as CT scans and MRI or fluoroscopy. 3D cameras' high resolution images provide the surgeons with superior visual clarity that supports precise procedures within restricted surgical areas, including tissue manipulation and organ extraction. The da Vinci Surgical System, alongside other robotic systems, uses 3D imaging to improve depth perception for prostatectomy and cardiac surgeries [14].

Pre-Operative Planning and Simulation

Imaging technologies function as vital tools during the period preceding the beginning of the surgical operation. The prior use of CT or MRI scans enables medical personnel to generate precise 3D models that guide surgeons through their planning process. The model can be integrated into robotic systems to direct surgical movements. Medical practitioners benefit substantially from imaging-guided surgery when performing colorectal surgery because it enables them to navigate procedures without damaging sensitive organs and tissues​ [2].

Haptic Feedback

A result of the advancements in robotic systems, the haptic feedback feature allows surgeons to experience tissue resistance and texture while operating. The robotic arms transmit sensory feedback, helping to recreate the sense of touch that is absent in traditional minimally invasive surgery [7].

Navigation and Imaging System

Introducing integrated navigation and imaging systems in real time, such as fluoroscopy, CT, MRI, and 3D ultrasound, has significantly enhanced the accuracy of RAS. The surgical system provides ongoing updates on internal structure positions and conditions, helping surgeons perform procedures without complications. Advanced surgical software integrates multiple imaging systems to develop 3D patient-anatomy models that are accessible before and during surgery and also track their progress [2].

Robotic Platforms

Computers have been developed for specialized surgical platforms through robotic designs. The da Vinci Surgical System has become a popular platform for assisting surgeons in general, urological, and gynecological medical procedures. The Versius system serves multiple surgical specialties, featuring modular adaptability for colorectal, urological, and gynecological procedures [19]​.

Targeted Therapy via Advanced Robotic Imaging System

The development of robotic systems has led to improved functionality with AI-powered imaging systems that autonomously detect crucial human body elements. Robotic arms work in conjunction with these systems to perform specific medical procedures that execute biopsies and tumor elimination tasks with enhanced precision. These technologies may eventually synchronize with nanorobots and microrobots, guided by sophisticated imaging systems, to perform precise procedures at the molecular and cellular level [2]​.

Multiple technological advancements have made surgical procedures less invasive, leading to enhanced patient recovery after surgery, reduced postoperative pain, and fewer surgical complications. However, the implementation of robotic surgery faces challenges, such as equipment costs and the need for further development of physicians’ skills. The future development of surgical robots will focus on smaller dimensions and improvement of AI systems as well as the expansion of autonomous surgical functions [20].

Comparison of prominent robotic systems

Various robotic systems have become important in general surgery, increasing surgical accuracy and visual observation and enabling minimally invasive treatment options. The three most popular robotic systems are the da Vinci Surgical System (Mountain View, CA: Intuitive Surgical, Inc.), the Versius Surgical Robotic System (Cambridge, UK: CMR Surgical Ltd.), and the Hugo RAS System (Minneapolis, MN: Medtronic). These systems provide a wide range of general surgical procedures, using state-of-the-art technological systems to assist physicians during various procedures, including colorectal surgery, cholecystectomy, and hernia-repair interventions. A comparison of these surgical systems examining technical and operational capabilities are discussed below [13].

da Vinci Surgical System

Intuitive Surgical operates its general surgery robotic system under the name da Vinci Surgical System (Mountain View, CA: Intuitive Surgical, Inc.). This robotic system provides flexibility, allowing medical teams to perform colorectal surgery, cholecystectomy, and hernia repairs with better outcomes. The robotic system contains three or four robotic arms, which enhance surgical dexterity through expanded movement capabilities beyond traditional laparoscopic tools. Surgeons operate specialized surgical tools such as scissors, staplers, and cauterizers using the console, with tools that are located inside the robotic arms [10].

The da Vinci system delivers 3D visualization capabilities and high-definition imaging as its primary advantage because it provides superior depth perception to traditional 2D laparoscopic surgery. Enhanced tissue clarity, achieved via improved visualization, allows medical staff to perform precise movements with greater accuracy in restricted areas. System feedback enables surgeons to sense resistance during procedures, which enhances both accuracy and surgical execution in addition to tissue manipulation performance [19].

The da Vinci system provides healthcare institutions with two primary benefits, although manufacturers must resolve particular implementation challenges. The adoption of this system by large medical facilities is becoming difficult due to its specialized nature, which leads to high system costs and training expenses. Medical studies have shown that although the system improves surgical accuracy, the operating duration is longer than standard laparoscopic procedures [14].

Versius Surgical Robotic System

Cambridge Medical Robotics (CMR) developed the Versius, which provides better modular units than the da Vinci system (Cambridge, UK: CMR Surgical Ltd.). Versius demonstrates flexibility in a wide range of procedures, including colorectal, urological, and gynecological procedures. The seven degrees of motion freedom allow robots to navigate difficult anatomical areas with improved precision and dexterity.

Users can modify the Versius system’s modular framework through its intuitive setup interface to meet various clinical requirements. The robotic system provides better ergonomics to surgical teams by offering flexible operating room functions and adjustable robot arms that adapt to specific surgical requirements. The surgical guidance technology delivers rapid patient healing together with key operational improvements enabled by 3D-HD image monitoring and advanced panoramic imaging functional capabilities [19].

The Versius system has gained market popularity because it offers lower costs than the da Vinci system while seamlessly integrating with standard operating room equipment. The system is seeing growing acceptance for general surgical procedures including colorectal resections and hernia repairs, providing hospitals with an affordable robotic surgical solution [1].

Hugo RAS System

The Hugo RAS robotic system, developed by Medtronic Inc. (Dublin, Ireland), functions as a general surgery robotic system. It serves multiple surgical purposes, including colorectal surgery, hernia repairs, and gallbladder removal. Surgeons operate the Hugo robotic system through its robotic arms from a console to achieve precise surgical outcomes, similar to the da Vinci and Versius systems [18].

The Hugo system provides a task simulator that allows surgeons to develop surgical skills before operating. This training tool helps reduce learning challenges connected with robotic surgery and demonstrates its ability to enhance surgical skills. The 3D visualization from the Hugo system gives healthcare providers better control while operating on complex anatomical structures and handling tissue precisely during surgery​ [18].

The Hugo system has demonstrated potential benefits to surgical outcomes and shorter patient recovery times, and its market acceptance continues to grow. The Hugo system stands out as a cost-efficient alternative to the da Vinci platform, making it suitable for hospitals operating on tight budgets. However, like other robotic platforms, the system requires substantial financial commitments for personnel training and infrastructure setup [8,18].​

The da Vinci Surgical System stands out among robotic surgery platforms because it is the most widely implemented platform due to its efficient functionalities and enhanced visualization capabilities. However, the Versius minimally invasive robotic system and the Hugo RAS system offer notable alternatives, particularly in terms of cost-effectiveness and modular flexibility. The Versius system offers surgical settings versatility due to its adaptable design and ergonomic features, and the Hugo system provides a task simulator for surgeon training, reducing learning-curve challenges. The choice among robotic surgical platforms depends on several factors, such as budget constraints, team training needs, and the specific requirements of surgical procedures. Advanced robotic systems are likely to increase the precision, safety, and positive outcomes in general surgery (Table 2).

**Table 2:** Comparison of different robotic systems based on their key characteristics and capabilities.

Features	Da Vinci Surgical System [10,14,19]	Versius Surgical Robotic System (minimally invasive) [1,19]	Hugo RAS System [8,18]
Manufacturer	Intuitive Surgical, Inc. (Mountain View, CA)	CMR Surgical Ltd. (Cambridge, UK)	Medtronic, Inc. (Minneapolis, MN)
Number of robotic arms	3-4 robotic arms	3 robotic arms (modular and flexible)	3-4 robotic arms
Degrees of freedom (DoF)	7 DoF per arm	7 DoF per arm	6-7 DoF per arm
Key surgical areas	General surgery, urology, gynecology, cardiac, colorectal, etc.	Colorectal, urological, gynecological surgeries	Colorectal, general surgery, hernia repairs, gallbladder surgery
3D visualization	Yes, high-definition 3D view	Yes, 3D-HD visualization	Yes, 3D visualization
Haptic feedback	Yes, provides tactile feedback	No haptic feedback	No haptic feedback
Modular design	No	Yes, modular and reconfigurable	No
Ergonomics	Surgeon seated at a console with intuitive controls	Modular arms for better ergonomics and easier setup	Surgeon seated at a console with intuitive controls
Training simulator	No	No	Yes, task simulator for training
Cost	High	Lower cost than da Vinci	Lower cost than da Vinci
Platform flexibility	Limited flexibility suited for most procedures	Highly flexible and adaptable for different procedures and room setups	Somewhat flexible but less modular than Versius
Clinical adoption	Widespread, considered the gold standard in robotic surgery	Growing adoption, particularly in Europe	Growing, but not as widely adopted as da Vinci
Robotic arms design	Rigid, but highly precise	Modular and flexible, adaptable for different procedures	Rigid, precise, but with fewer degrees of freedom than Versius
Primary advantage	Precision, versatility, and advanced 3D visualization	Portability, flexibility, and adaptability in various settings	Affordability, ease of integration, and task simulator for skill enhancement

Current applications of robotic surgery to improve clinical outcomes

Conventional Versus Robot-Assisted Minimally Invasive General Surgeries

The general surgery realm has been transformed by MIS because it decreases the need for extensive incisions, thus reducing pain and speeding up recovery times. Robotic surgery helps a surgeon obtain increased precision, superior visualization, and enhanced control, making RAS optimal for complex surgeries. Robotic surgery and conventional MIS are demonstrating reduced invasiveness, improved precision during surgery, and accelerated recovery times, based on recent research findings, particularly for patients and surgeons.

Invasiveness reduction: The main goal of MIS is to make small incisions, thus decreasing trauma to nearby tissues, minimizing blood loss, and lowering the risk of infection. Robot-assisted MIS enables surgeons to make smaller incisions due to better hand dexterity and 3D visualization compared with conventional MIS. In a 2025 meta-analysis, Bobade and Asutkar compared robotic-assisted minimally invasive esophagectomy (RAMIE) and conventional laparoscopic esophagectomy (cMIE), confirming that robotic surgery leads to smaller incision size and reduced intraoperative blood loss. The study revealed robotic surgery reduced blood loss by 71.78 mL (p<0.00001) compared with laparoscopic surgery [21]. In another study, Wehrle et al. in 2024 reported that robotic pancreaticoduodenectomy surgery with the Whipple procedure resulted in a lower need for open surgery (p<0.05) due to smaller incisions and fewer postoperative complications [22]. Robotically-assisted surgical platforms enable surgeons to execute complex procedures while reducing invasion compared with conventional surgical openings.

Improvement in precision: A set of robotic arms attached to a robotic system provides surgeons with better manual control for precise surgical tasks by increasing dexterity. The high 3D visualization of robotic systems also helps surgeons increase depth perception, enhancing tissue handling and structure identification with greater accuracy. Hancock et al. in 2022 found that robotic colorectal resections produced less blood loss than laparoscopic surgeries - the robotic group experienced 55.2±29.8 mL blood loss, less than the laparoscopic group’s 109.5±58.5 mL (p<0.05)​ [17]. Robotic surgical instruments give operators precise control over dissection, thus decreasing the risk of bleeding into the body and preventing surgical complications. The precise placement of mesh in hernia repair surgeries is made possible by robotic-assisted techniques, which decrease hernia reoccurrence rates. The research comparing robotic and laparoscopic hernia repairs showed that robotic systems performed mesh placement more accurately than laparoscopic systems, leading to less hernia recurrence and superior long-term outcomes​ [23]. The advanced precision of robotic systems generates better clinical outcomes, lowering complication rates and reducing conversions to open surgery.

Enhanced recovery times: Robotic surgery patients recover more rapidly because robotic surgery decreases the traumatic effects that are typically linked to conventional open surgery. Shortening procedure times helps to decrease the chances of postsurgical infections, decreasing acute pain levels and promoting earlier discharge from the hospital. Less blood loss and better surgical precision accelerate tissues’ recovery, enabling patients to recover and return to their presurgical activities more quickly. Recovery of gastrointestinal systems occurs quickly in robotic colorectal surgical procedures. The time required for the first flatus after robotic colorectal resections was also shorter than after laparoscopic surgery, according to the study by Hancock et al. in 2022 [17]. Patients who underwent robotic gastrointestinal procedures achieved first flatus within 35.1±9.4 hours, faster than laparoscopic surgery patients, who took 40.7±1.9 hours (p<0.05), according to ​the study by Bobade and Asutkar in 2025 [21]. In another study of 2024, Wehrle et al. performed robotic pancreaticoduodenectomies and noted that robotic surgery yielded a 3.03-day shorter hospital stay than laparoscopic surgery (p<0.0001), demonstrating faster recovery from robotic operations. Hernia repair with robotic technology yielded faster recovery with less pain and earlier patient mobility compared with standard open surgery [22]. The data show robotic surgery to be effective in decreasing postoperative recovery duration. Table 3 presents and compares the current applications of robot-assisted general surgeries with conventional surgeries.

**Table 3:** Comparison of robotic and conventional general surgeries outcomes. *Conventional surgery stands for open surgery or surgeries without robotic assistance.

Surgery	Robot-assisted surgery	Conventional surgery*
Colorectal surgery	Reduced blood loss by 71.78 mL (p<0.00001), shorter recovery, fewer complications, reduced conversion to open surgery [17]	Higher blood loss (109.5±58.5 mL), longer recovery, increased complications, higher conversion rates [17]
Hernia repair	Lower recurrence rates, reduced complication rates, faster recovery by one to two days, smaller incisions [14]	Higher recurrence rates (up to 10-15%), increased complications, longer recovery, larger incisions [14,24]
Pancreatoduodenectomy	Shorter hospital stays by 3.03 days (p<0.0001), reduced blood loss, better lymph node yield, fewer complications [22]	Longer hospital stays, more blood loss, fewer lymph nodes harvested, higher complication rates (25-30%) [22]
Cholecystectomy	Reduced conversion rates by 1.7%, faster recovery, improved precision, fewer postoperative complications [8,25]	Higher conversion rates by 3%, longer recovery, increased risk of complications (5-10%) [8]
Esophagectomy	Shorter recovery by 3.03 days, fewer pulmonary complications, improved precision, lower conversion rates [16]	Longer recovery (avg. three days), more pulmonary complications (5-10%), lower precision, higher conversion rates [16]

Robotic-assisted general surgery: advantages and disadvantages for patients and surgeons

Clinical Advantages for Patients

Robotic surgery provides several clinical advantages, such as better clinical outcomes, fewer postoperative complications, and rapid recovery rates. The rate of complications decreased in RASs due to better control, precision, and shorter duration of surgery. The research indicates robotic colorectal surgery reduces blood loss with fewer conversions to open surgery and has lower rates of infection than traditional approaches. Robot-assisted general surgery also produces smaller scars while offering less pain to patients and speeding up recovery. According to a 2022 study by Hancock et al., patients who benefit from robotic gastrointestinal surgery recover faster, spend less time in the hospital, and return to daily activities and pass flatulence more quickly [17].

Disadvantages for Patients

However, RAS has some disadvantages. The expenses associated with robotic surgery surpass those of traditional laparoscopic or open surgeries, even though robotic surgery sometimes leads to better outcomes. Healthcare facilities raise their procedure costs and hospital fees to cover the increased expenses associated with robotic surgery. Patients encounter substantial financial challenges to robotic surgery when their healthcare systems are weak or insurance coverage does not extend to robotic procedures. Furthermore, although robotic systems function reliably, technical equipment breakdowns and system malfunctions remain possible risks. A malfunctioning robot during surgery may cause delays to the procedure, which may force surgeons to perform open surgery, leading to increased complications and longer recovery times. Patient safety is put at risk when these circumstances occur, particularly when system failures occur at crucial times. Operating times may be longer when surgeons are first learning robotic surgery because of lack of experience. Although surgeons’ experience improves performance with time, longer operating times can extend patient anesthesia duration and increase their risk of complications, especially in elderly or high-risk patients. Robotic surgery remains unavailable in many healthcare facilities, especially in low-resource clinical settings. Patients in these areas need to travel to specialized medical facilities for treatment [2].

Advantages for Surgeons

The primary benefit robotic surgery delivers to surgeons, particularly in complex surgeries, is its high degree of precision and control. Among robotic systems, the da Vinci Surgical System delivers advanced high-definition 3D visualization that enables surgeons to view their surgical site with much greater visual clarity than with conventional procedures. The system includes tremor-filtering and movement-scaling functions, allowing precise execution of tiny, delicate tasks with remarkable precision. These capabilities are useful for complex procedures, including colorectal resections and pancreaticoduodenectomy, in which meticulous motor movements are vital. Furthermore, robotic systems provide surgeons with an ergonomic advantage that enhances their work experience. Conventional open surgical procedures force surgeons to maintain painful body positions for long periods that may cause musculoskeletal strain. The robotic system enables surgeons to conduct procedures from a console while seated, resulting in a substantial reduction of physical or musculoskeletal strain. Robot-assisted surgery enables surgeons to maintain sharp focus for long surgical durations and provides comfort by reducing physical fatigue in surgeons and helping them work efficiently. Robotic surgery decreases surgeons’ physical workload by removing the need for manual handling of instruments, robotic suturing, tremor reduction, and robotic surgery assistance. High-dexterity and multistep procedures, such as gastrointestinal surgeries and pancreaticoduodenectomy, benefit significantly from these features. The rate of improvement in performance is noticeable after the initial learning phase, and operative durations decrease with experience. Hancock et al. in 2022 also demonstrated that surgeons develop enhanced efficiency through practice during colorectal resections [17]. The mastery of robotic systems results in a reduction in operation time duration which shortens the total length of the procedure. Improved efficiency reduces both the time surgeons spend on complex surgeries and the time patients spend under anesthesia.

Disadvantages for Surgeons

However, the initial learning for robotic systems demands extensive practice and long training periods that might extend surgical times, particularly for inexperienced surgeons. Robotic systems may provide insufficient haptic feedback to surgeons, so they struggle with tissue-resistance detection during surgical maneuvers, thus increasing risks during procedures. The prolonged use of the console with robotic systems causes mental exhaustion for surgeons who need to simultaneously operate multiple systems while maintaining their concentration. The expense of robotic systems exceeds $2 million and includes yearly maintenance costs exceeding $100,000, which restricts their availability to hospitals operating on tight budgets. Surgeons develop technical dependency, but these systems malfunction, which forces them to switch between robotic and conventional surgical operations, thus creating possible risks during life-threatening circumstances [14,26].

Challenges and limitations

Various limitations are associated with robotic surgery methods, which pharmaceutical companies should understand in addition to their beneficial aspects.

Cost

The availability of robotic systems in hospitals is limited due to the high acquisition, operating, and maintenance costs, which are manageable only by wealthier medical institutions. The purchase of da Vinci systems requires an initial investment exceeding $2 million and annual maintenance expenses of approximately $100,000​ [17,26].

Initial Learning

Medical professionals need extensive robotic-surgery training to achieve proficiency; initial robotic operations typically take additional time and yield varied outcomes​ [26].

Haptic Feedback

The absence of complete haptic feedback from robotic systems reduces surgeons’ ability to detect tissue resistance and other touch-based surgical signals during operations [22,26].

Clinical implications

The adoption of robotic surgery has generated major changes in general surgery practices, affecting both patient outcomes and surgical performance. Robotics systems enhance the precision of complex operations, producing better outcomes that minimize patient complications, reduce healing time, and improve the cosmetic appearance of wounds. RAS has several main advantages, such as smaller wounds that promote quick recovery, less pain, and shorter duration of hospitalization. Surgical efficiency improves as surgeons become experts at robotic systems, enabling faster operations and demonstrating enhanced surgical skills, which optimize operating room time. Robotic surgery is poised to redefine conventional treatment approaches in general surgery while simultaneously improving healthcare efficiency and patient satisfaction as it becomes increasingly user-friendly.

Way forward and recommendations

Robot-assisted surgery may advance as solutions are found for current challenges and more clinical implementation of robotic systems occurs. A competitive manufacturers’ environment, combined with technological progress, will reduce robotic system costs, making these systems more affordable. The integration of AI technology into robotic systems would enable better surgical decisions, reduce operator learning requirements, and increase procedural accuracy. Standardized training consists of developing standardized robotic-surgery training programs and simulators to establish consistent and efficient surgeon proficiency.

Currently, RAS exists with robots either executing preprogrammed tasks or gaining experience through deep-learning models (DLMs), which process feedback from good and bad outcomes. Artificial neural networks (ANNs) represent the digital version of biological nervous systems. The development of autonomous robots begins with the creation of a DLM using ANN technology. The development of independent robots requires four essential tools as follows: 2D surgical scene segmentation, depth-chart reconstruction, surgical-skill evaluation, and surgical simulation and planning [27,28]. AI delivers algorithms that endow machines with opinion-forming and cognitive-processing abilities, which could define the future of surgical robotics [28]. Future surgical training systems will likely rely on machine learning through automated performance metrics that observe surgeons’ techniques to determine surgical assessments before making predictions to benefit real-time medical decisions. The current technology does not show evidence that AI can discover key robotic surgical tasks affecting patient results [29]. The medical field requires immediate investigation into extensive datasets while validating the external application of AI algorithms. RAS surgical systems with higher levels of autonomy could achieve standardized results independent of the surgeons’ training backgrounds, experience, or daily performance variations. Survival tests have demonstrated that the developed robotic system matched expert surgeon capabilities regarding leak-free anastomosis and lumen patency results, exhibiting better consistency.

Despite initial reports in published literature, the use of RAS in emergency situations remains unexplored [30]. The research needs to continue to fully comprehend the robotic surgery effects on long-term outcomes and develop better practices for general surgery. Researchers must follow patients over time to determine how robotic surgery affects their health outcomes, cancer survival rates, quality of life, and healthcare expenses. It is important to conduct comparative analyses among robotic surgery, laparoscopic surgery, and open surgery. Such comparisons will help determine cost effectiveness, clinical benefits, and patient satisfaction measurements. Integrating AI with robotic systems can enhance real-time decision-making to improve surgical outcomes. Therefore, future researchers should focus on the integration of AI with robotics. Research must explore protocols and methods related to the learning curve, which aim to help surgeons effectively adopt robotic-surgery procedures while maintaining patient safety. Research into these topics will optimally use RAS across general surgery practice as the field continues to develop.

## Conclusions

Robot-assisted surgery in general surgery has brought significant changes to multiple aspects of surgical practice, as reflected in the existing literature. The primary research outcomes show that robotic surgery minimizes surgical invasiveness by enabling smaller incisions, decreasing blood loss, and accelerating patient recovery. The da Vinci Surgical System has shown enhanced surgical accuracy due to improved control when operating in complex cases such as colorectal resections, pancreatoduodenectomy, and hernia repairs. The use of robotic systems enables surgeons to remain comfortable while performing complex procedures because these systems allow them to maintain ergonomic body positions. Robotic systems lead to better clinical patient outcomes, with patients experiencing shorter hospital stays, lower complication rates, and rapid recovery times. However, the full advantages of robotic surgery will only emerge once technical challenges, such as higher costs, longer initial learning curves, and haptic feedback issues, are adequately and comprehensively addressed.

## References

[1] Biswas P, Sikander S, Kulkarni P (2023). Recent advances in robot-assisted surgical systems. Biomed Eng Adv.

[2] Dagnino G, Kundrat D (2024). Robot-assistive minimally invasive surgery: trends and future directions. Int J Intell Robot Appl.

[3] Qian L, Wu JY, DiMaio SP, Navab N, Kazanzides P (2019). A review of augmented reality in robotic-assisted surgery. IEEE Trans Med Robot Bionics.

[4] Cepolina F, Razzoli RP (2022). An introductory review of robotically assisted surgical systems. Int J Med Robot.

[5] VINCI D: surgical system, Intuitive Surgical (2025) Da Vinci X: a foundational entry point to robotic surgery. https://www.intuitive.com/en-us/products-and-services/da-vinci/x.

[6] Krebs TF, Kayser T, Lorenzen U (2023). Evaluation of the Versius robotic system for infant surgery - a study in piglets of less than 10 kg body weight. Children (Basel).

[7] Liu Q, Zhang W, Zhao JJ (2023). Propensity-score matched and coarsened-exact matched analysis comparing robotic and laparoscopic major hepatectomies: an international multicenter study of 4822 cases. Ann Surg.

[8] Perry R, Barbosa JP, Perry I, Barbosa J (2024). Short-term outcomes of robot-assisted versus conventional minimally invasive esophagectomy for esophageal cancer: a systematic review and meta-analysis of 18,187 patients. J Robot Surg.

[9] Gharagozloo F, Tempesta B, Meyer M, Nguyen D, Gruessner S, Redan J (2021). History of robotic surgery. Robotic Surgery.

[10] Morrell AL, Morrell-Junior AC, Morrell AG, Mendes JM, Tustumi F, De-Oliveira-E-Silva LG, Morrell A (2021). The history of robotic surgery and its evolution: when illusion becomes reality. Rev Col Bras Cir.

[11] Liu Y, Wu X, Sang Y, Zhao C, Wang Y, Shi B, Fan Y (2024). Evolution of surgical robot systems enhanced by artificial intelligence: a review. Adv Intell Syst.

[12] Jayaraman S, Davies W, Schlachta CM (2009). Getting started with robotics in general surgery with cholecystectomy: the Canadian experience. Can J Surg.

[13] Asadizeidabadi A, Hosseini S, Vetshev F, Osminin S, Hosseini S (2024). Comparison of da Vinci 5 with previous versions of da Vinci and Sina: a review. Laparosc Endosc Robot Surg.

[14] Rivero-Moreno Y, Echevarria S, Vidal-Valderrama C (2023). Robotic surgery: a comprehensive review of the literature and current trends. Cureus.

[15] Maggard-Gibbons M, Girgis M, Ye L (2020). Robot-Assisted Procedures in General Surgery: Cholecystectomy, Inguinal and Ventral Hernia Repairs. https://pubmed.ncbi.nlm.nih.gov/34038044/.

[16] Lunardi N, Abou-Zamzam A, Florecki KL (2024). Robotic technology in emergency general surgery cases in the era of minimally invasive surgery. JAMA Surg.

[17] Hancock KJ, Klimberg VS, Nunez-Lopez O, Gajjar AH, Gomez G, Tyler DS, Rashidi L (2022). Optimizing outcomes in colorectal surgery: cost and clinical analysis of robotic versus laparoscopic approaches to colon resection. J Robot Surg.

[18] Sheetz KH, Claflin J, Dimick JB (2020). Trends in the adoption of robotic surgery for common surgical procedures. JAMA Netw Open.

[19] AlSajri AA, Hariry N (2024). The future of surgery: robotic-assisted procedures and their impact. SHIFAA.

[20] Picozzi P, Nocco U, Labate C (2024). Advances in robotic surgery: a review of new surgical platforms. Electronics.

[21] Bobade S, Asutkar S (2025). Current trends and future directions in surgery: a brief scoping review. Mult Rev.

[22] Wehrle CJ, Chang JH, Gross AR (2024). Comparing oncologic and surgical outcomes of robotic and laparoscopic pancreatoduodenectomy in patients with pancreatic cancer: a propensity-matched analysis. Surg Endosc.

[23] Reddy K, Gharde P, Tayade H, Patil M, Reddy LS, Surya D (2023). Advancements in robotic surgery: a comprehensive overview of current utilizations and upcoming frontiers. Cureus.

[24] See CW, Kim T, Zhu D (2020). Hernia mesh and hernia repair: a review. Eng Regen.

[25] Sakpal SV, Bindra SS, Chamberlain RS (2010). Laparoscopic cholecystectomy conversion rates two decades later. JSLS.

[26] Cruz EM, Oliveira S, Correia A (2024). Robotics application in the hospital domain: operator-patient-robot interface. [Preprint]. Preprints.org.

[27] Bhandari M, Zeffiro T, Reddiboina M (2020). Artificial intelligence and robotic surgery: current perspective and future directions. Curr Opin Urol.

[28] Moglia A, Georgiou K, Georgiou E, Satava RM, Cuschieri A (2021). A systematic review on artificial intelligence in robot-assisted surgery. Int J Surg.

[29] Saeidi H, Opfermann JD, Kam M (2022). Autonomous robotic laparoscopic surgery for intestinal anastomosis. Sci Robot.

[30] Arang H, El Boghdady M (2023). Robotic appendicectomy: a review of feasibility. Sultan Qaboos Univ Med J.

